# Omni-OTPE: Omnidirectional Optimal Real-Time Ground Target Position Estimation System for Moving Lightweight Unmanned Aerial Vehicle

**DOI:** 10.3390/s24051709

**Published:** 2024-03-06

**Authors:** Yi Ding, Jiaxing Che, Zhiming Zhou, Jingyuan Bian

**Affiliations:** The School of Automation Science and Electrical Engineering, Beihang University, Beijing 100083, China; dy990517@163.com (Y.D.);

**Keywords:** unmanned aerial vehicle (UAV), target position estimation, fisheye camera, LiDAR, data fusion

## Abstract

Ground target detection and positioning systems based on lightweight unmanned aerial vehicles (UAVs) are increasing in value for aerial reconnaissance and surveillance. However, the current method for estimating the target’s position is limited by the field of view angle, rendering it challenging to fulfill the demands of a real-time omnidirectional reconnaissance operation. To address this issue, we propose an Omnidirectional Optimal Real-Time Ground Target Position Estimation System (Omni-OTPE) that utilizes a fisheye camera and LiDAR sensors. The object of interest is first identified in the fisheye image, and then, the image-based target position is obtained by solving using the fisheye projection model and the target center extraction algorithm based on the detected edge information. Next, the LiDAR’s real-time point cloud data are filtered based on position–direction constraints using the image-based target position information. This step allows for the determination of point cloud clusters that are relevant to the characterization of the target’s position information. Finally, the target positions obtained from the two methods are fused using an optimal Kalman fuser to obtain the optimal target position information. In order to evaluate the positioning accuracy, we designed a hardware and software setup, mounted on a lightweight UAV, and tested it in a real scenario. The experimental results validate that our method exhibits significant advantages over traditional methods and achieves a real-time high-performance ground target position estimation function.

## 1. Introduction

The value of lightweight unmanned aerial vehicle (UAV) applications continues to increase with new developments in control systems and sensing systems. With the advantages of small size and flexible operation, lightweight UAVs can operate in cluttered and narrow environments, which is more conducive to the execution of ground target reconnaissance and search tasks in near-earth scenarios and is widely used in urban scene monitoring [1,2,3], wilderness searching and locating [4,5,6], and other application scenarios requiring real-time target detection and localization [7,8,9]. Among them, in order to improve the detection efficiency, the use of panoramic sensors can obtain all the ground environment information at a certain moment and can realize the function of real-time output of target position information to improve the utilization value of information.

Since lightweight UAVs have limited carry capacity, target detection and localization methods based on lightweight UAVs can be divided into image information-based methods, point cloud information-based methods, and fusion-based methods.

Image information-based methods mainly rely on visual sensors to acquire real-time scene images and perform image target detection to find the target image location, and then solve for the position information of the target in the real scene. Compared to a standard pinhole camera, a fisheye camera has a field of view greater than 180 degrees, allowing panoramic images of the ground scene to be acquired in real time. However, fisheye camera images have large radial aberrations, which can lead to performance degradation of traditional feature-based target detection algorithms [10,11]. Therefore, the current target detection based on fisheye images usually adopts the target detection method based on deep learning [12,13]. Among them, you only look once (YOLO) [14] combines both detection accuracy and detection speed, which is suitable for limited embedded computing platforms with limited computational resources [15,16,17]. The methods for target localization based on fisheye images can be classified into projection process-based methods and deep learning-based methods. The basic principle of the method based on the projection process is to solve the position information of the corresponding point in the real scene of the image target center point according to the fisheye camera imaging model combined with the known absolute dimension information in the scene, considered as the position of the target [18,19]. Due to the low complexity and faster computation speed of this method, it can be run in real time on lightweight UAV computing platforms [20]. However, limited by the detection accuracy as well as the absolute size measurement error, the effective detection distance of this method is about 2 m, which limits the scope of use. The deep learning-based approach uses a neural network structure to construct a relationship between the detected target image and the true position of the target [21]. The key is to extract the edge information [22] or semantic feature information [23] of the target in the current image and combine it with the trained model for position estimation. It can be deployed on lightweight platforms to achieve real-time position estimation [24,25,26]. The maximum effective detection distance of the current deep learning-based methods is about 10 m performance due to the traditional methods. However, the system performance in real deployment is related to the quality of the training set, and the target effective features also affect the accuracy of the target position estimation.

The method based on point cloud information mainly relies on the environmental point cloud information obtained from distance sensors such as LiDAR [27] or Radar [1] for processing, extracts the effective point cloud cluster of the target according to the target features, and uses the coordinates of the center of the point cloud cluster as the center coordinates of the target. The core of the method is how to find the target point cloud clusters in the point cloud map. The traditional method is to construct a classifier based on the shape features of the target and detect the target point cloud in the sensed point cloud image and then get the position information [27,28], but this method relies on the shape features of the target and can not be, respectively, similar to the shape of the object. Recent approaches using deep learning have been validated to extract more implicit information about the target and enhance target detection accuracy [29,30] but are still inherently dependent on the target’s shape features and require the use of high-performance LiDAR sensors to acquire high-density point cloud maps [31]. However, these devices are heavy and difficult to use on lightweight UAV platforms. Currently, along with the development of sensor lightweight technology, the application of lightweight LiDAR sensors on UAVs is increasing [32,33], but the application in target identification and detection is yet to be developed.

Since the advantages and disadvantages exhibited by the above two methods show complementarity, sensor fusion can be used to improve the system performance, which is the idea of execution based on the fusion method. Sensor fusion methods can be categorized into a variety of techniques: early fusion, late fusion and intermediate fusion [34]. Early fusion methods merged raw or low-level preprocessed data to produce high-quality raw data but increased the computational effort of the solution [35]. The late fusion method used a fuser [36,37] to obtain a better result after obtaining two results. Intermediate fusion can be understood as a combination of the first two techniques, merging data at multiple levels to get better data, but it increases the complexity of the system [38,39]. Currently, this method is usually applied to autopilot [40,41] as well as stationary detection scenarios [42], with fewer UAV-based solutions yet to be developed.

**Figure 1:**
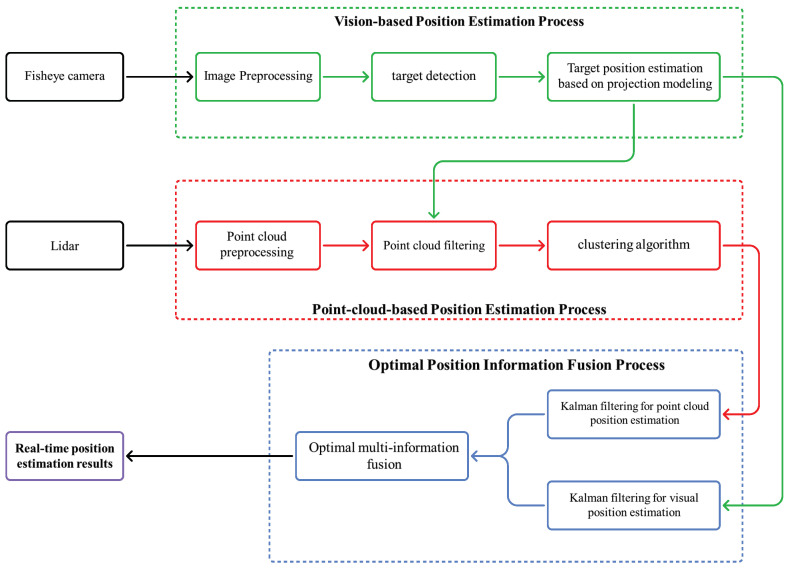
Omni-OTPE: Omnidirectional optimal real-time ground target position estimation system framework.

**Figure 2:**
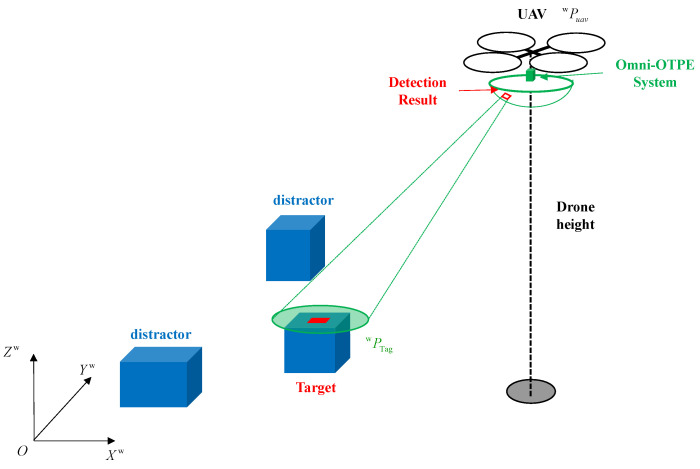
The drone is equipped with Omni-OTPE system that allows it to estimate the position of a target-tagged object in conjunction with its own position.

In this paper, an omnidirectional optimal real-time ground target position estimation system (Omni-OTPE) is proposed, which can be deployed on an embedded platform to sense the ground environment omnidirectionally in real time and output the detected target position, as shown in Figure 1. Our system will be divided into three processes: Image-based position estimation, the point cloud-based position estimation and optimal target position fusion. Image-based position estimation focuses on a fisheye camera for collecting image data of the scene. Target detection is performed on the original fisheye image and a real-time target position estimate is obtained by running a target center-point position-estimation system based on the spherical coordinate system based on the recognition frame edge information. Point cloud-based position estimation focuses on a LiDAR for collecting point cloud data of the scene. By using a position–direction-based point cloud filtering approach, redundant points are removed. After that, a point cloud clustering approach is used to provide accurate target localization results. Due to the different working principles, the two localization results differ in rate and accuracy. Therefore, the Kalman fusion filter is used to obtain the optimal target location information. In addition, in order to test the system performance, we propose a set of hardware and software solutions for a ground target sensing system that can be deployed with our system and test them in a physical environment to verify the localization performance, as shown in Figure 2. In this case, we deploy three objects with similar appearance characteristics, of which only the labeled object is our target object, and our system needs to detect the labeled object and output its position information under the global coordinate system in real time.

This paper’s major contributions are as follows:

- We provide a method for estimating the position of a target center using edge information detection. This algorithm reduces the error between the extracted target center and the real value, thus improving the accuracy of position estimation;
- We propose an efficient position–direction point cloud screening method that utilizes visual localization results to effectively exclude unnecessary information from the point cloud and improve the accuracy of target location extraction from the point cloud;
- We present a position estimator which utilizes a Kalman fusion filter to get optimal results. This estimator merges two types of localization information using the mutual covariance matrix of the position estimation error. The result is a target position estimation that is both real-time and precise;
- We design an efficient hardware and software solution for precisely estimating the position of a target. It allows us to install our system on a lightweight unmanned aerial vehicle (UAV) and prove the efficiency of our method in a real-world scenario.

The rest of this paper is organized as follows: Section 2 briefly describes our system; Section 3 demonstrates the image-based position estimation algorithm; Section 4 demonstrates LiDAR-based positioning optimization algorithm. Section 5 demonstrates the Kalman filter-based multi-information fusion technique. Section 6 shows the experimental results, while some conclusions are given in Section 7.

## 2. System Overview

**Figure 3:**
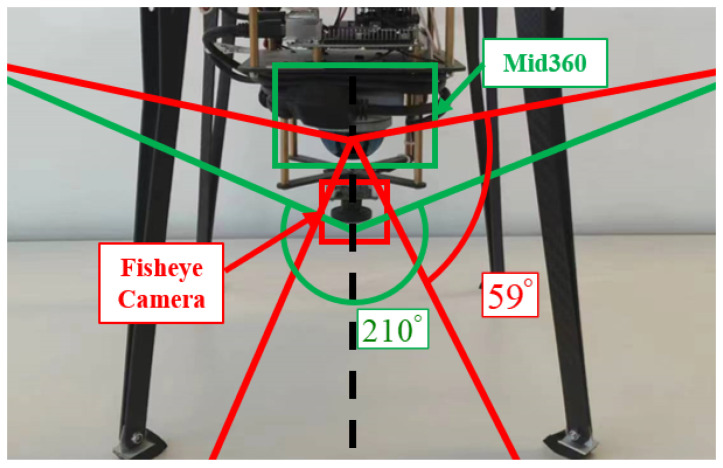
The layout of the sensors and the range of vertical sensing angles.

For real-time omnidirectional target position estimation, we chose to use two wide-angle environmental sensors: a fisheye camera as well as a LiDAR. The fisheye camera utilizes the AR0237 digital image sensor from the US-based onsemi manufacturer (Phoenix, AZ, USA), which outputs images at a frequency of 20 Hz, and the point cloud sensor is a Mid360 LiDAR sensor made by Livox in Shenzhen, China, which can operate at 15 Hz and generate 200,000 points per second. The layout of the sensors and the range of vertical sensing angles are shown Figure 3. The horizontal sensing angle range of these two sensors is $360^{\circ }$, and the effective sensing range can be up to 20 m, which can meet the general needs of ground detection. The computing platform uses an ARM-based embedded lightweight computing platform with a Jetson Orin NX module from Nvidia, Santa Clara, CA, USA, which can use version 8.5.2 of TensorRT to accelerate the speed of yolov5 image recognition and improve the algorithm operation in real time. The proposed method was implemented in C++ using the Noetic version of the Robot Operating System (ROS) on an on-board computer with Ubuntu 20.04 system to output the position of the target under the global coordinate system in real time at a speed of 10 Hz. Our target position estimation hardware system weighs about 700 g and can be mounted on a lightweight UAV with a wheelbase of 350 mm. Combined with a global satellite positioning-based UAV control system, it can acquire the position information of the UAV in real time and improve the target positioning accuracy.

The definitions of the variables that appear in the text are shown in the Table 1.

## 3. Image-Based Position Estimation Process

Image is widely used as information for target detection and localization because of its benefits of high spatial resolution, dense data structure, and high information entropy. However, standard pinhole cameras possess a limited visual range, thus posing difficulties with promptly finding targets within broad environments using a single camera. For this reason, we use a fisheye camera, which can obtain an omnidirectional image in real time, which is conducive to improving the efficiency of target detection.

At first, the target detection method is applied to obtain the target detection results on the original fisheye image. Next, a spherical coordinate system is used to depict the transformed correlation between the image points and their corresponding points in reality. The algorithm for estimating the position of the target center point utilizes the detected edge information to obtain an estimate of the target’s position based on the image data.

### 3.1. Fisheye Camera Target Detection

We adopt YOLOv5 [43], one of the state-of-the-art visual object detection approaches based on a convolutional neural network (CNN), for detecting the 2D bounding boxes of the target on the distortion-free images extracted from raw fisheye images. The network is trained with our custom data to efficiently detect our targets.

**Figure 4:**
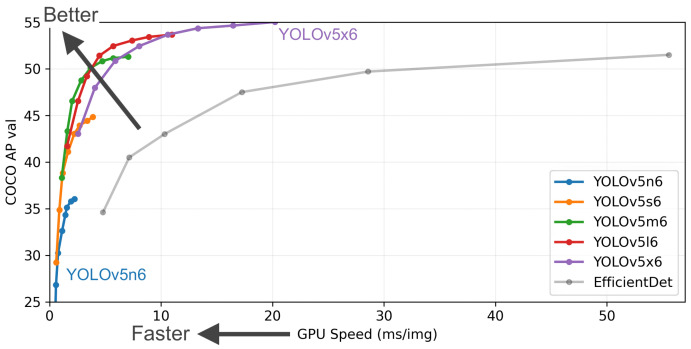
Yolov5 performance plots for each model.

In addition, there are multiple variants of yolov5, each of which has differences in speed and accuracy under different application conditions, and the optimal model needs to be tested according to the scenario. The official performance results of each model run given by yolov5 [44] are shown in Figure 4.

### 3.2. Target Position Estimation Based on Fisheye Camera Model

**Figure 5:**
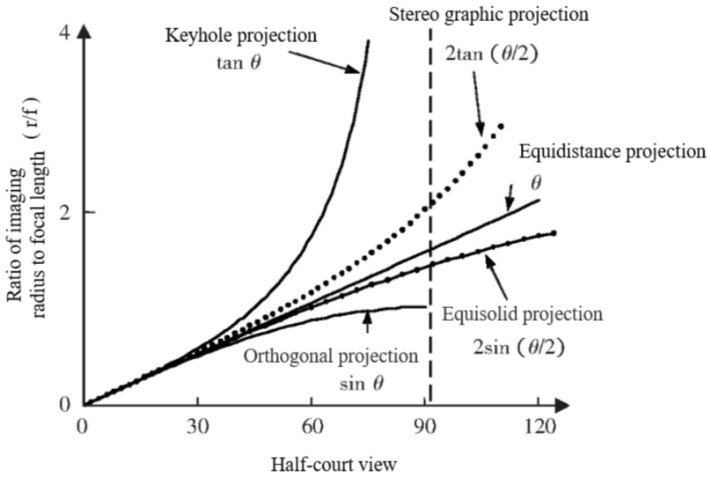
Comparison of distortion of different projection models. The model includes Keyhole projection, Stereo graphic projection, Equidistance projection, Equisolid projection and Orthogonal projection. Pinhole projection is the standard camera model, which theoretically has no distortion. The degree of distortion of the model can be expressed as the degree of curve deviation between this model and pinhole projection model.

To determine the relationship between detected box information and the real-world position of an object, it is essential to acquire the polynomial equations of the fisheye camera model. As shown in Figure 5, the imaging principle of the fisheye camera is different from that of the standard pinhole camera, resulting in the projection model of the fisheye camera being closer to the Equidistance projection model, which leads to an increase in the degree of aberration of the fisheye image accompanied by an increase in the angle of incidence. In addition, it is difficult to establish a common model expression due to the differences in the production process, lens combination and other factors of different fisheye cameras. This leads to difficulties in representing the projection model of the fisheye camera with an accurate expression. Reference [45] proposes using a general polynomial approximation model to establish the model relationship, and the five odd degree polynomials can express the imaging relationship of the model well. It can be expressed as

(1) $$r=m_{0}\theta +m_{1}\theta^{3}+m_{2}\theta^{5}+m_{3}\theta^{7}+m_{4}\theta^{9}$$

where θ denotes the angle of incidence of the light and *r* denotes the pixel distance from the imaging point in the image to the center of the image. The coefficients $m_{j}$ can be obtained from camera calibration. In our case, *d* is obtained by detector, and θ can be obtained from Equation (1) by solving a root of a nonlinear algebraic equation problem. The fixed-point iteration is adapted to obtain the solution of the equation. In order to carry out the method, Equation (1) is transformed into the following expression:

(2) $$\theta =\frac{r}{m_{0}+m_{1}\theta^{2}+m_{2}\theta^{4}+m_{3}\theta^{6}+m_{4}\theta^{8}}.$$

By iterating Equation (2) several times, an accurate solution for θ can be obtained.

To date, we can use the center of the camera image as the origin to construct a spherical coordinate system $\rho^{V}=\left[\varphi ,\theta ,l\right]$, where φ is the azimuth angle, which can be solved by the coordinate relationship using the inverse sine function; θ is the polar angle solved by Equation (2). Due to the lack of depth information in the monocular image, the module length *l* information in the spherical coordinate system is difficult to obtain directly and needs to be combined with the absolute size information in the actual scene in order to get the accurate value.

In order to improve the efficiency of pattern length computation, we make the following assumptions in conjunction with the inspection scenario illustrated in Figure 2:

- There are valid target images in the image with fisheye projection models that accurately represent the polar angle information at different pixel locations.
- The terrain in the reconnaissance area is flat and the upper surface of the target is parallel to the ground.
- The detection algorithms can accurately recognize the label pattern on the target, and the detection box in the inspection result can correctly characterize the limit size information of the label.

From this, it is possible to solve for the position of the target center point using the real-time height of the UAV as an absolute dimension.

**Figure 6:**
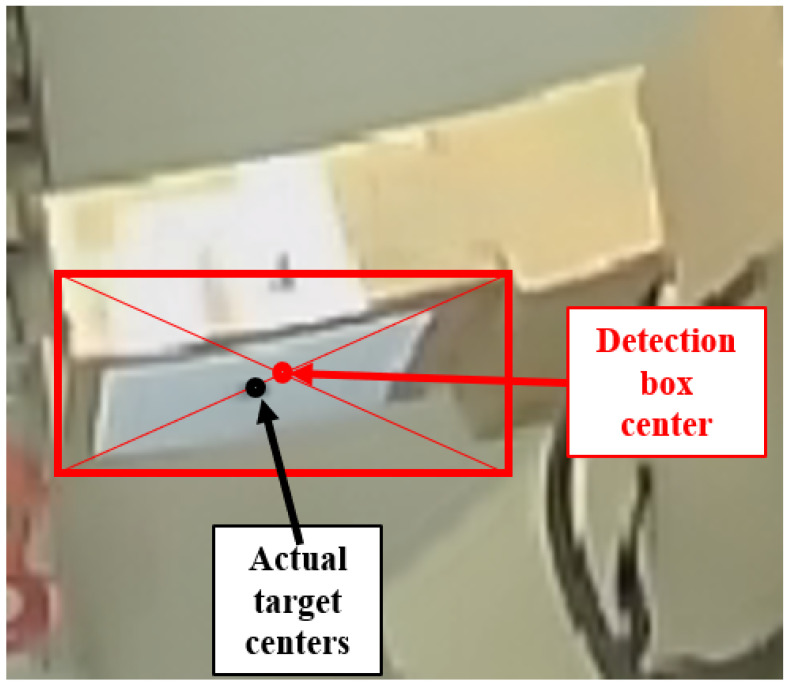
Description of the center offset problem.

Traditional fisheye-based camera-position estimation algorithms typically use the center position information of the detection frame to solve for the target centroid through a fisheye projection process model. However, due to the presence of aberrations in the fisheye image, this can lead to a difference between the corresponding point of the target center in the image and the center point of the detection box, as shown in Figure 6.

**Figure 7:**
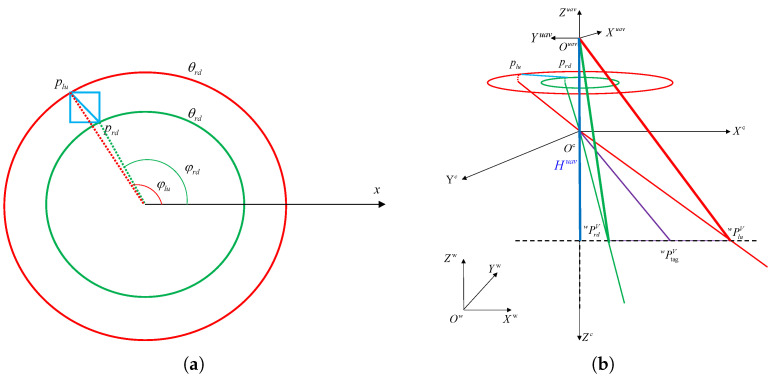
(**a**) Polar and azimuthal angles corresponding to the upper left (red) and lower right (green) fixed points of the identification box in the fisheye image. (**b**) Fisheye camera point projection process.

To this end, we propose a new target center-point position-estimation method based on the detected edge information, which can effectively improve the localization efficiency by solving the points corresponding to the upper-left and lower-right points of the frame in the actual scene through the model, and solving the target center point position through the midpoint equation, as shown in Figure 7.

To obtain the position of the target midpoint, we need to solve for the coordinates of the upper left and lower right points of the detection frame in the world coordinate system, as shown in Figure 7a. Each of these points has a corresponding polar angle and azimuth angle. Therefore, we can solve the Cartesian coordinates of the two points under the camera coordinate system according to the principle of coordinate system transformation, and the generalized formula is

(3) P∗Vc=l∗sinθ∗cosφ∗l∗sinθ∗sinφ∗l∗cosθ∗

where ${\left(.\right)}_{*}$ represents the pixel point to be solved; P∗Vc denotes the Cartesian coordinates of the pixel point in the camera coordinate system; $\rho_{*}^{V}=\left[\varphi_{*},\theta_{*},l_{*}\right]$ denotes the coordinates of the spherical coordinate system of the corresponding pixel point.

As shown in Figure 7b, in order to obtain the coordinates of the points in the world coordinate system, it is also necessary to transform the coordinates using the following equations:

(4) P∗Vw=TuwTcuP∗Vc=X∗VwY∗VwZ∗Vw

where P∗Vw denotes the position of the pixel point under the world coordinate system; P∗Vc denotes the position of the pixel point under the camera coordinate system; $T_{u}^{c}$ denotes the transformation relation from the UAV coordinate system to the world coordinate system, which can be obtained based on the current positional attitude of the UAV; $T_{c}^{u}$ denotes the transformation relationship from the camera coordinate system to the UAV coordinate system, which can be obtained by the calibration method.

According to the assumed conditions, the lower left and upper right points of the detection box can be considered to be in the same plane, and the straight line constructed by the two points is parallel to the ground, which can be expressed as

(5) ZluVw=wZrdV=Huav−hTag

where Z∗Vw denotes the height information of a point in the world coordinate system; $H^{uav}$ is information about the current altitude of the drone and can be used with the current altitude of the UAV; $h_{Tag}$ denotes the compensation value of the target height, which is initially 0. Considering that there is a position difference between the target and the ground, $h_{Tag}$ can be updated with the altitude information from the back-end target repositioning. Combined with Equations (3)–(5), we can get the depth information $l_{*}$. Considering the adverse effects of misdetection, we also include a constraint handling process. When the calculated depth information $l_{*}$ is greater than the maximum detection distance (15 m) or less than the minimum detection distance (1 m), we consider it as a false detection and reject it. From this, we can get information about the coordinates of the upper-left point PluVw and the lower-right point PrdVw. The position of the center of the target PTagVw is calculated as follows:

(6) PTagVw=PluVw+wPrdV2

Because of the non-ideal conditions in real scenarios, the vision-based position estimates deviate significantly from the true values, as the estimation model for module lengths is not accurate. However, this approach is highly efficient in terms of processing speed and may rapidly acquire approximate position information about the target.

## 4. Point Cloud-Based Position Estimation Process

To enhance the precision in estimating the target’s position, we perform target localization on the real-time point cloud provided by the LiDAR. A position–orientation-based point cloud filter is applied to the filtered point cloud to identify the point clouds near the visual localization. A clustering algorithm is next used to obtain the point clouds that match the target. This process offers the target’s position information based on the point cloud data.

### 4.1. Point Cloud Pre-Processing

Since the points in the real-time point cloud are obtained by laser ranging, the real-time point cloud also needs to be preprocessed in order to obtain the position of the target point in the world coordinate system, denoted as

(7) piw=TuwTlupil

where pil denotes the coordinate information of a point in the original point cloud data; piw is the point coordinate in the set of point clouds under the LiDAR coordinate system; $T_{l}^{u}$ denotes the bit position information of the current state of the UAV; and $T_{l}^{u}$ denotes the LiDAR sensing its transformation matrix into the UAV coordinate system, which can be obtained by sensor calibration. Due to the limited spatial point attributes that can be expressed by point cloud information, carrying out target detection and localization directly in point cloud maps consumes a large amount of computational resources and is difficult to run in real time on embedded platforms. Therefore, we refer to the visual target position estimation information. In order to align the time frames, a delay compensation method is needed to solve the target position under the current frame, and this process is expressed as

(8) PTagtVw=wPTagV+KVuavw

where PTagtVw denotes the estimate of visual position after time alignment; *K* denotes the delay factor; and Vuavw denotes the current speed of the UAV.

**Figure 8:**
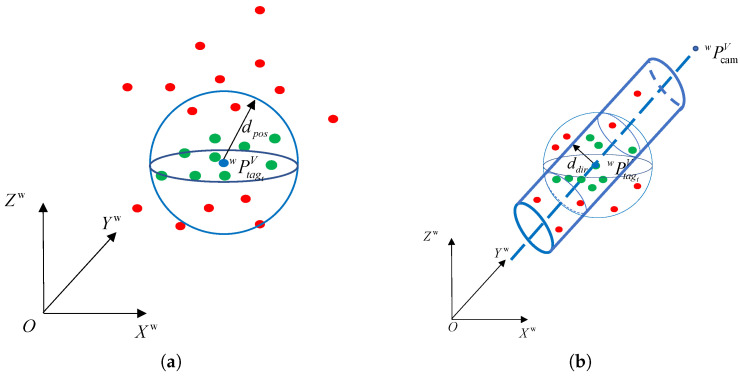
(**a**) Position-based point cloud filtering. (**b**) Direction-based point cloud filtering, where the green dots are the point cloud generated by the target; the red dots indicate anomalies; and the blue dots indicate vision-based position estimates PtagVw.

The number of points in the point cloud set at the current moment affects the target localization accuracy, but too many point clouds can slow down the operation. To solve this problem, we process the acquired point cloud data using a point cloud filter, which filters the global point cloud $\Theta_{w}$ based on the front-end visual position estimation point information. In Figure 8, the filtering rules are as follows:

- Position-based point cloud filtering: Based on the target size, the points with distance to the visual position less than the position filtering threshold $d_{pos}$ are filtered to form a new point cloud set $\Theta_{pos}$, as shown in Figure 8a. Specifically, the point $p_{i}^{pos}$ in the point cloud set $\Theta_{pos}$ satisfy the relation
  (9) $$|\vec{P_{Tag_{t}}^{V}p_{i}^{pos}}|\leq d_{pos}$$
- Direction-based point cloud filtering: The error in height estimation is the main reason for localization accuracy, and the polar angle as well as azimuthal angle measurements are relatively accurate in comparison, so the orientation-based point cloud filtering is used to filter the point cloud set $\Theta_{pos}$. Constructing vector PcamVwPTagtVw→, the points with distance to PcamVwPTagtVw→ less than the direction filtering threshold $d_{dir}$ are recorded as valid points, and a new point cloud set $\Theta_{dir}$ can be obtained, as shown in Figure 8b. Specifically, the point $p_{i}^{dir}$ in the point cloud set $\Theta_{dir}$ satisfy the relation
  (10) PcamwPTagtw→×Pcamwpidir→Pcamwpidir→≤dpir

In summary, position-based filtering uses vision-based position estimation information to quickly filter out point cloud information near the target. However, due to the large error in the vision-based position estimation information, there are still more invalid point cloud information in the obtained point cloud set $\Theta_{pos}$. For this reason, we use orientation-based filtering to further remove the number of invalid point clouds by using the more reliable target orientation information in the visual position estimation to improve the positioning performance of the subsequent target relocation algorithm.

### 4.2. Target Relocalization

After obtaining the set of point clouds where the target exists, a point cloud clustering algorithm can be used to obtain the set of target point clouds. The FLANN-based kd-tree point cloud clustering search algorithm [46] is used to carry out the clustering of the point cloud set using the Euclidean distance, and after obtaining multiple clustering results, the point cloud set closest to the visual localization point PtagVw is regarded as the target, and the computation can be used to obtain the LiDAR-based target location PtagLw.

The point cloud data output from LiDAR is highly accurate, but due to the limitation of the field of view, the position of the target cannot be observed throughout the whole process.

## 5. Optimal Position Information Fusion Based on Kalman Filtering

Combining the performance of the two sensors, it can be seen that visual localization can quickly measure the position of the target, but the localization accuracy is low; point cloud target localization has a high localization accuracy, but the point cloud generation speed is slow and limited by the field of view angle, resulting in poor real-time performance. For this case, the two information points can be fused using a Kalman fuser to obtain the best position estimate that combines the advantages of both methods.

**Figure 9:**
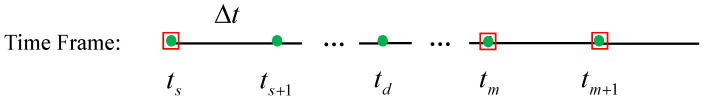
Time series of the target location output.

**Figure 10:**
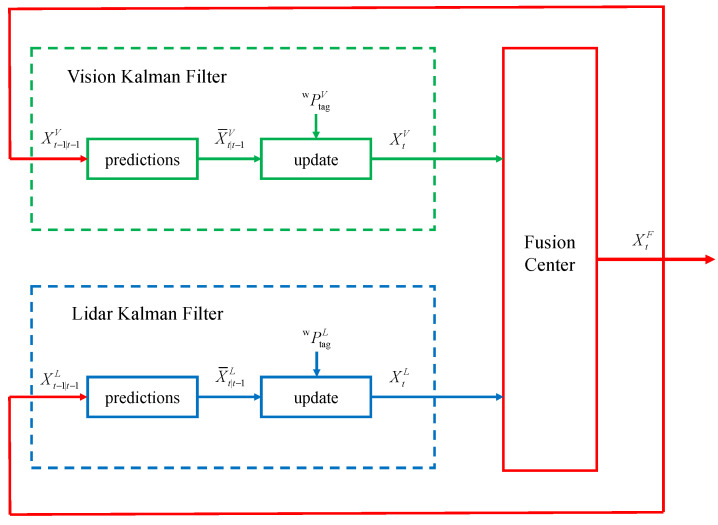
Optimal target position information fusion process.

To obtain the optimal target position estimation, we use a Kalman filter to obtain high-quality real-time target position information. Firstly, the time distribution of each part of the system needs to be analyzed, as shown in Figure 9. The green dots are the output frames of visual localization; when the label of the target is detected, the visual localization system can quickly get the position value of the target and output it; the red box indicates the output frames of LiDAR localization, due to the existence of the visual blind area of the LiDAR, which results in the visual localization results not finding the matching point cloud cluster, and the localization results can not be guaranteed to be output in real time. Since the time alignment operation is performed in the point cloud processing, it can be regarded as the point cloud information acquired at the same moment with the visual localization information. However, since LiDAR-based target position estimation cannot be output in real time. Therefore, we constructed a new information fusion system as shown in Figure 10.

In a frame, the vision and LiDAR enter two Kalman filters (KF), respectively. For static targets, the estimated state quantities are denoted as

(11) X∗=Ptag∗w

Kalman filters can be constructed. Where the prediction stage equation is expressed as

(12) $$\begin{array}{cc} \; & {\bar{X}}_{t|t-1}^{*}=AX_{t-1}^{*}\\ \; & {\bar{P}}_{t|t-1}^{*}=AP_{t-1}^{*}A^{T}+Q \end{array}$$

where the state transfer matrix *A* as well as the process noise covariance matrix *Q* are the same for both methods since both measurements directly estimate the position information of the target.

The update phase is expressed as

(13) $$\begin{array}{cc} \; & K_{t}^{*}={\bar{P}}_{t|t-1}^{*}F_{t}^{T}{\left(F_{t}{\bar{P}}_{t|t-1}^{*}F_{t}^{T}+R^{*}\right)}^{-1}\\ \; & X_{t}^{*}=X_{t}^{*}+K_{t}\left(z_{t}^{*}-F{\bar{X}}_{t|t-1}^{*}\right)\\ \; & P_{t}^{*}=\left(I-K_{t}^{*}F_{t}\right){\bar{P}}_{t|t-1}^{*} \end{array}$$

where $F_{t}$ is the measurement matrix, which is the same for both methods, and $R^{*}$ is the measurement noise covariance matrix. This leads to the vision-based Kalman filter state $X_{t}^{V}$ and the LiDAR-based Kalman filter state $X_{t}^{L}$.

To get the optimal state $X_{t}^{F}$, the following step is to combine the two sets of data. Reference [47] provided a comprehensive optimal estimation fusion solution for cases where two estimates exhibit correlation. The fused estimate can be obtained by combining two estimates, $X_{t}^{V}$ and $X_{t}^{L}$, which have estimating error covariance matrices $P_{t}^{L}$ and $P_{t}^{V}$, respectively.

(14) $$\begin{array}{cc} X_{t}^{F} & =X_{t}^{L}+\left(P_{t}^{L}-P_{LV}\right){\left(P_{t}^{L}+P_{t}^{V}-P_{LV}-P_{VL}\right)}^{-1}\left(X_{t}^{V}-X_{t}^{L}\right)\\ P_{t}^{F} & =P_{t}^{L}-\left(P_{t}^{L}-P_{LV}\right){\left(P_{t}^{L}+P_{t}^{V}-P_{LV}-P_{VL}\right)}^{-1}\left(P_{t}^{L}-P_{VL}\right) \end{array}$$

$P_{LV}={P_{VL}}^{T}$ is the cross-covariance matrix of estimations errors. Combined with the modified delayed track to track fusion (MDTTF) method proposed in [48], the cross-covariance matrix can be solved using the predicted values of the vision-based KF and the LiDAR-based KF at the current moment, denoted as

(15) $$P_{VL}=AP_{t-1}^{F}A^{T}+\left(I-K_{t}^{L}F\right)Q-\left(I-K_{t}^{V}F\right)Q\left(AK_{t}^{L}F\right)$$

Combining Equations (13) and (14), the fused state $X_{t}^{F}$ and covariance $P_{t}^{F}$ can be obtained.

When the LiDAR does not detect a target, the LiDAR-based filter does not output an estimate. At this point, if it is in the initial state, it can directly output the visual position. Instead, the LiDAR-based position state at the current moment can be obtained by prediction by combining the latest point cloud position information in the past.

As shown in Figure 9, at moment $t_{d}$, since there is no location estimate based on the point cloud, it is necessary to use the prediction equation to obtain the state of ${\bar{X}}_{t_{d}}^{L}$ at the current moment as well as the covariance matrix ${\bar{P}}_{t_{d}}^{L}$. This can be expressed as

(16) $$\begin{array}{cc} {\bar{X}}_{t_{d}}^{L} & ={\left(A\right)}^{d-s}X_{t_{s}}^{L}\\ {\bar{P}}_{t_{d}}^{L} & ={\left(A\right)}^{d-s}P_{t_{s}}^{L}{\left(A^{T}\right)}^{d-s}+\sum_{l=1}^{d-s}{\left(A\right)}^{l-1}Q{\left(A^{T}\right)}^{l-1} \end{array}$$

where $X_{t_{s}}^{L}$ and $P_{t_{s}}^{L}$ denote the state and covariance matrix at the output moment of the latest point cloud position estimate before moment $t_{d}$. As a result, the fused position estimate can be obtained, which combines real-time as well as localization accuracy and can output high-quality target position estimates.

## 6. Experimental Results and Discussion

To evaluate the system’s capacity to precisely estimate the target’s position, we proceed by designing a test environment in a real-world environment and executing flight experiments. At first, a calibration step is conducted for each sensor to gain the necessary characteristics. Then, a target detection model that fulfills the specified criteria is chosen based on rigorous testing. Finally, we carry out flying experiments in actual circumstances to assess the system’s ability to precisely estimate the position of the target.

### 6.1. Sensor Calibration

The use of multi-sensor systems requires calibration of sensor-related parameters to minimize the adverse effects caused by parameter errors. Parameters to be calibrated include

1. Fisheye camera distortion parameters $m_{i}$: The polynomial coefficient parameters of Equation (1) are obtained based on the method proposed by [45].
2. Transformation matrix from fisheye camera coordinate system to UAV coordinate system $T_{c}^{u}$: After setting up the known transformation relation, the fisheye camera is used to localize the target at a known position in space, and the error function is constructed, and the transformation matrix is updated to get the exact matrix using the Gaussian–Newton method.
3. Transformation matrix from LiDAR coordinate system to UAV coordinate system $T_{l}^{u}$: Similar to the calibration method for $T_{c}^{u}$. Target detection of point clouds can be improved by placing highly reflective material on the target.

### 6.2. Performance on Target Detection

In order to set up an effective recognition model, it is necessary to generate a collection of fisheye images that include the target of interest. The label of interest in the actual scenario is an image measuring 24 × 24 cm. When launching the UAV and positioning it in close proximity to the target, we can obtain fisheye images of the UAV from different positions in real time. This allows us to get images from several perspectives, resulting in enhancing the accuracy of detection.

To select the optimal model, we trained three Yolo-v5 models separately to compare their accuracy as well as the computational time, and the results are shown in Table 2.

The dataset data consist of fisheye images, each containing target imaging at different positions from the camera. After manual labeling, they are entered into yolov5 model training. The training parameters as well as the results are shown in Table 2. The yolov5-m model accurately recognizes the position of the target in the image with a mean detection accuracy (mAP) of 0.926, which is the highest accuracy among the three models. In addition, the model can be run on an on-board computer at a speed of about 20 Hz, which satisfies the real-time requirement.

**Figure 11:**
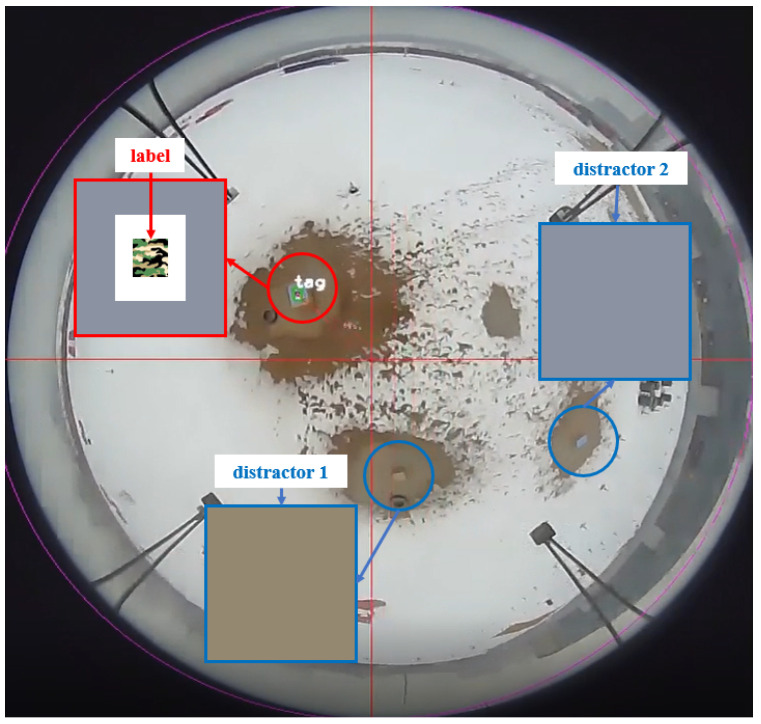
Scene detection graph. There are three box objects of similar size in the scene. The one with the gray surface and label is the target.

The test results in real-world scenarios are shown in Figure 11. This indicates that our algorithm can stably detect specially labeled targets.

### 6.3. Target Localization Experiments

**Figure 12:**
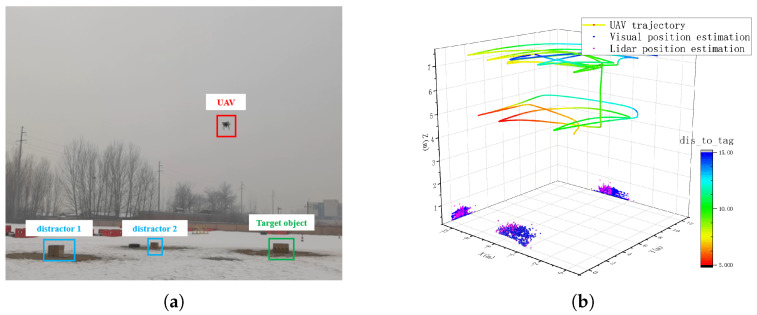
(**a**) Flight experiment in which there are two distractors and one target object; (**b**) UAV trajectory and target position estimates, where the color of the trajectory can indicate the distance from the UAV to the target.

We bring in the trained yolov5-m model and conduct target localization experiments based on the localization method proposed in this paper. First, we build the experimental scenario as shown in Figure 12a, where the UAV moves randomly at an altitude of 5 m and 7 m, respectively, the system detects the target at a frequency of 10 Hz and outputs the position of the target in the global coordinate system. The flight trajectory and target localization results are shown in Figure 12b. The farthest detection distance is 15 m. For this purpose, we set up three different types of boxes on the ground, and the items to be detected and localized are boxes with target images. To compare the localization accuracy, we use the real-time kinematic positioning system (RTK) to obtain the position information of each unit (including UAVs, target objects and jammers). Due to the robustness as well as the high localization accuracy of the RTK system, it can be used as a control group to test the localization performance. Their locations are shown in Table 3. Target object is the object to be detected.

### 6.4. Experiment of Image-Based Target Position Estimation

**Figure 13:**
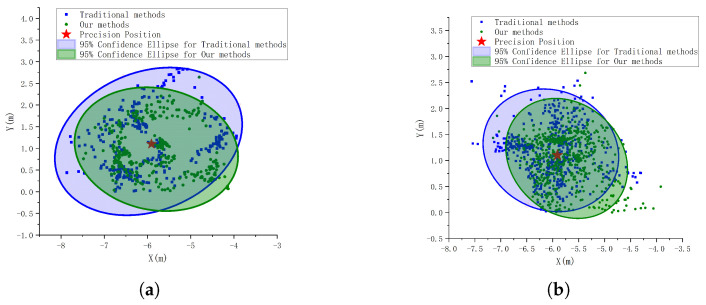
Image-based plot of horizontal coordinates of the estimated points of the target position, indicating the distribution of the estimated points. (**a**) Estimated target position at 5 m altitude by the UAV. where the ellipse denotes the 95 confidence ellipse, which can indicate the degree of distribution of the position estimates. (**b**) Estimated target position at 7 m altitude by the UAV.

In order to verify the advantages of our visual localization algorithm, the sensor data information in flight are recorded, and the traditional target localization method and our new visual visual localization method are run separately to localize the target in real time during two flight segments of 5 m and 7 m, and the target position estimation results are obtained as shown in Figure 13.

The distribution of estimated points is described using confidence ellipses with a confidence level of 95%. It can be found that the detection results of the two methods are around the target truth value, which indicates the feasibility of the methods. The distribution of the estimated points of the target position obtained by our method in the two sets of experiments is more concentrated and the center of the ellipse is near the target true value position, indicating that the target center position obtained by our method is more robust.

We use angular error to compare the advantages and disadvantages of the two target center position estimation algorithms. The angular error between the estimated value and the true value at each moment can be expressed as

(17) θerr=arcsinPcamwPTagEstw→×PcamwPTagTruew→PcamwPTagTruew→PcamVwPTagEstw→

where Pcamw denotes the UAV coordinates in the global coordinate system of the current frame; PTagEstw denotes the estimated position of the target in the global coordinate system of the current frame; and PTagTruew denotes the true position of the target in the global coordinate system of the current frame.

**Figure 14:**
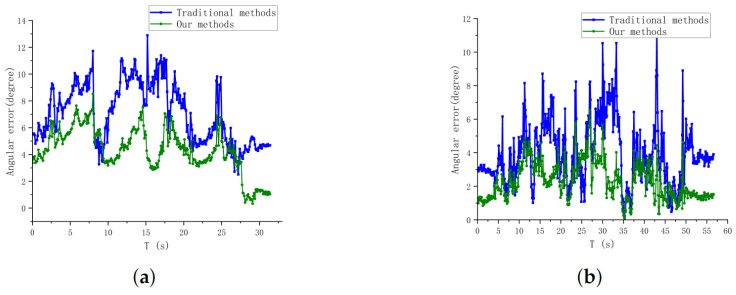
Direction angle error curves for both methods. (**a**) Angular error in position estimation in flight at 5 m altitude. (**b**) Angular error in position estimation in flight at 7 m altitude.

From this, the angular error $\theta_{err}$ of the two methods at different heights can be obtained, as shown in Figure 14. During the flight at 5 m altitude, the angular error of our method is smaller than that of the traditional method, with an average angular error of $4.331^{\circ }$, which is nearly 40% lower compared to the traditional method; however, during the flight at 7 m altitude, the angular errors of the two methods are similar due to the small area of the detected image. The average angular error of our method is $2.322^{\circ }$ degrees, and the traditional method is $3.941^{\circ }$ degrees. The results show that our method can effectively reduce the angular error of the localization system, and the closer the distance to the target, the smaller the angular error. In addition, since our method relies on the target edge detection information, it is more robust and reduces the number of times when a large error peak occurs compared to methods that use the center of the detection frame directly.

**Figure 15:**
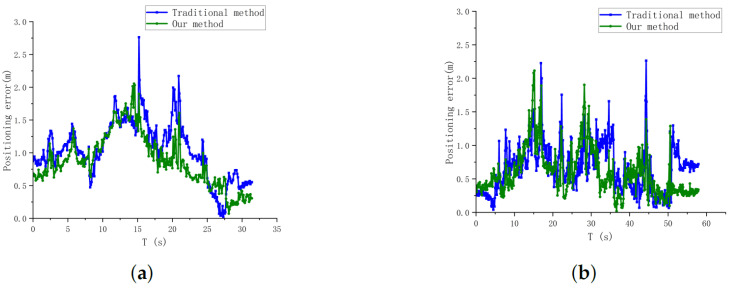
Target position estimation error curves for both methods. (**a**) Positioning error in position estimation in flight at 5 m altitude. (**b**) Positioning error in position estimation in flight at 7 m altitude.

**Figure 16:**
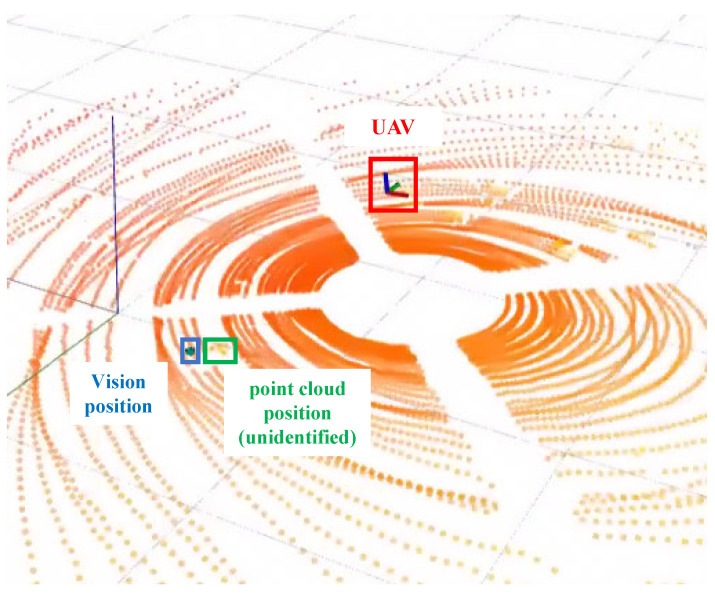
When the visual localization error is too large, the back-end will reject the correct point cloud information, reducing the back-end repositioning performance.

Finally, the positioning error curves of the two methods can be plotted according to the target position truth as shown in Figure 15. The comparison results show that our method can effectively reduce the localization error and reduce the number of peaks appearing in the figure, indicating that our method can reduce the target position error well. In addition, but when the distance increases, the small area of the detection frame leads to similar localization accuracy of the two methods. However, since our method is obtained from the target limit size, it is more robust and has fewer large-size errors compared to conventional methods. This is conducive to fewer occurrences of point cloud matching failure scenarios due to visual positional bias, as shown in Figure 16. In practical tests, our method successfully matches 214 times with point cloud targets in 5m flight experiments, compared to 184 times with traditional methods, and 408 times with point cloud targets in 7 m flight experiments, compared to 238 times with traditional methods. This shows that our target center extraction method can effectively improve the accuracy and robustness of target detection, which is conducive to improving the position estimation performance of the system. However, the ideal conditions in the fisheye fixation model are difficult to be fully satisfied in real environments, resulting in large fluctuations in the visual localization values and poor position estimation performance.

### 6.5. Experiment of Optimal Target Position Estimation

**Figure 17:**
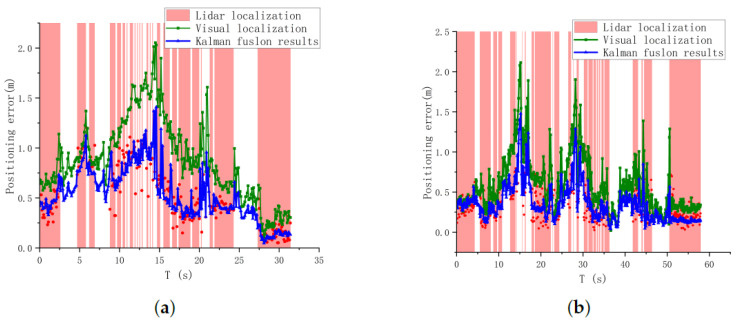
Three positional estimation error curves. (**a**) Target localization error of each localization method in 5 m flight experiment. (**b**) Target localization error of each localization method in 7 m flight experiment. The red point is the error of LiDAR localization, and the red area indicates that the system finds the corresponding target point cloud cluster at that moment based on the visual localization result. The blue curve is the position error curve after fusion.

In order to verify the advantages of our proposed optimal position estimation method, we compare the image-based position estimation, the point cloud-based position estimation, and the optimal position estimation with the target’s true position value at each moment to calculate the positioning error. According to the results of the image-based target position estimation error, the two parameters of the point cloud screening link in point cloud localization are set as $d_{pos}=1.5\;m,d_{dir}=1.0\;m$. The localization error per frame for the 5 m flight experiment as well as the 7 m flight experiment is shown in Figure 17.

The green curve indicates the localization results using only the fisheye camera. It can be found that although we optimized the visual localization method, it is difficult to fully conform to the ideal fisheye model in reality. The interference of external factors will cause the visual localization is not stable and the error fluctuation is large. While the method of using LiDAR to localize the target can yield a more accurate target position, LiDAR’s limited field of view and incorrect position matching can result in LiDAR not being able to achieve continuous output. Therefore, we fused the two position data to obtain the best position estimate that is both accurate and real-time, with the error curve in blue. As shown in Figure 17, in the two flight experiments, when the point cloud matching is successful (red area), the fused position error is less than the pure visual localization; even in the area where the matching is successful (white area), the fused position error is still less than the pure visual due to the a priori target position, which indicates that the information fusion can effectively reduce the error of visual localization.

Finally, we analyze the system in real time, and the specific elapsed time for each component is shown in Table 4. Since each link is relatively independent, we improve the execution efficiency of the program and reduce the computation time consumption by using parallel computation. Finally, our single-target position estimation computation consumes about 30 ms, and theoretically, we can output the positions of three targets simultaneously at a speed of 10 Hz.

## 7. Conclusions

This paper introduces a multi-sensor fusion-based ground target detection and localization technique, which effectively improves the robustness and localization accuracy of the detection and localization algorithm by performing target detection on unrecovered fisheye images and loosely coupling vision-based and LiDAR-based target localization results with UAV position information. We combine the principles of the algorithm to build a lightweight detection scheme that can be deployed on small UAVs with limited load capacity. Our experiments validate that the method can detect and localize targets in real time.

This algorithm mainly depends on the accuracy of the image detection algorithm, so the image detection in the algorithm requires more computational resources. The detection algorithm can be improved subsequently, which can further improve the accuracy of target localization.

## Figures and Tables

**Table 1:** Explanation of the meaning of important notations appearing in the text.

Notations	Explanation
${p_{rd}}^{\;1}$	The pixel coordinate corresponding to the lower right corner of the detection box.
${p_{lu}}^{\;1}$	The pixel coordinate corresponding to the upper left corner of the detection box.
${\theta_{rd}}^{\;1}$	The polar angle corresponding to the lower right corner of the detection box.
${\theta_{lu}}^{\;1}$	The polar angle corresponding to the upper left corner of the detection box.
${\varphi_{rd}}^{\;1}$	The azimuth angle corresponding to the lower right corner of the detection box.
${\varphi_{lu}}^{\;1}$	The azimuth angle corresponding to the upper left corner of the detection box.
${T_{u}^{c}}^{\;1}$	The transformation relation from the camera coordinate system to the UAV coordinate system.
$T_{u}^{w}$	The transformation relation from the UAV coordinate system to the world coordinate system.
$h_{Tag}$	The compensation value of the target height.
PrdVw 1	The estimated coordinate of the position of the center of the target in the world coordinate system.
PluVw 1	The estimated coordinate of the position of the center of the target in the world coordinate system.
${H^{uav}}^{\;1}$	Altitude values of the drone in the world coordinate system.
** PTagVw **	Target center position coordinates based on visual localization methods.
piw 2	The coordinate of the ith point in the LiDAR real-time point cloud based on the world coordinate system.
${T_{l}^{u}}^{\;2}$	The transformation relation from the LiDAR coordinate system to the UAV coordinate system.
pil 2	The coordinate of the ith point in the LiDAR real-time point cloud based on the LiDAR coordinate system.
PTagtVw 2	The estimated coordinates of the visual position after the alignment of two perceptual information time frames.
Vuavw 2	The UAV velocity vector.
$K^{\;2}$	Time compensation parameters.
${\Theta_{w}}^{\;2}$	The set of point clouds obtained by LiDAR.
${\Theta_{pos}}^{\;2}$	The set of point clouds obtained after the position filter.
${p_{i}^{pos}}^{\;2}$	The coordinate of the ith point in the set of $\Theta_{pos}$.
${\Theta_{dir}}^{\;2}$	The set of point clouds obtained after the direction filter.
${d_{pos}}^{\;2}$	Maximum distance condition in position filter.
${p_{i}^{dir}}^{\;2}$	The coordinate of the ith point in the set of $\Theta_{dir}$.
${d_{dir}}^{\;2}$	Maximum distance condition in direction filter.
** PtagLw **	Target center position coordinates based on point cloud localization methods.
${X^{*}}^{\;3,*}$	Filter state vector.
${P^{*}}^{\;3,*}$	Filter state covariance matrix.
$A^{\;3}$	Filter state transition matrix.
$Q^{\;3}$	Filter process noise covariance matrix.
${z_{t}^{*}}^{\;3,*}$	Filter state observation vector.
$F^{\;3}$	Filter observation matrix.
${R^{*}}^{\;3,*}$	Filter measured noise covariance matrix.
${{\bar{X}}_{t|t-1}^{*}}^{\;3,*}$	Filter state prediction vector.
${{\bar{P}}_{t|t-1}^{*}}^{\;3,*}$	Filter covariance matrix between true and predicted values.
${K_{t}^{*}}^{\;3,*}$	Filter gain matrix.
${P_{LV}}^{\;3}$	Covariance matrix of LiDAR-based localization and vision-based localization states.
** PtagFw **	Target center position coordinates based on optimal target position estimation.

^1^ Notations are only used in the image-based target position estimation process. ^2^ Notations are only used in the point cloud-based target position estimation process. ^3^ Notations are only used in the optimal target position estimation process. ^*^ Generic notations, with references to “∗” for specific meanings.

**Table 2:** Performance of different yolov5 models on embedded platforms.

Model	Number of Data	Iteration Step	mAP@IoU0.5 ^1^	Runtime ^2^
yolov5-n	1200	800	0.896	38.656 ms
yolov5-s	1200	800	0.862	34.862 ms
yolov5-m	1200	800	0.926	46.726 ms

^1^ mAP@IOU0.5 indicates mean average precision (IoU = 0.5). ^2^ Running time of the algorithm on an embedded computer (Jetson Orin NX).

**Table 3:** The true value of the position of each object in the scene in the world coordinate system.

Object Type	X (E ^1^)/m	Y (N ^2^)/m	Z (U ^3^)/m
Target	−5.910	1.101	0.496
Distractor 1	4.204	−5.476	0.458
Distractor 2	−4.257	−5.447	0.425

^1^ (E) East. ^2^ (N) North. ^3^ (U) Upward.

**Table 4:** Calculation of time consumed by each process.

Process	Gain Image	Gain Point Cloud	Visual Position Estimation	Lidar Position Estimation	Optimal Fusion Filter
Runtime ^1^	about 50 ms	about 100 ms	about 20 ms	about 35 ms	about 30 ms

^1^ The average time of the operations in each part of the experiment.

## Data Availability

The data presented in this paper are available after contacting the corresponding author. As these data are also part of an ongoing study, they are not publicly available.
